# P85 - Asthmatic children and adolescents treated in daily medical practice – results from a 2-year sublingual allergen immunotherapy (AIT) study with grass pollen tablets

**DOI:** 10.1186/2045-7022-4-S1-P140

**Published:** 2014-02-28

**Authors:** Efstrathios Karagiannis, Kija Shah-Hosseini, Meike Hadler, Ralph Mösges

**Affiliations:** 1 Stallergenes GmbH, Kamp-Lintfort, Germany; 2 Institute of Medical Statistics, Informatics and Epidemiology (IMSIE), University Hospital Cologne, Cologne, Germany

## Background

The aim of this non-interventional study was to document the impact of a sublingual allergen immunotherapy (AIT) with Oralair 5-grass pollen tablets (Stallergenes, France) on symptom severity (rhinitis, conjunctivitis, asthma), use of symptomatic medication and tolerability in patients with grass pollen-induced allergic rhinoconjunctivitis (RC) over 2 years of routine medical practice treatment. This poster focuses on the subgroup of asthmatic children (4-11 yrs) and adolescents (12-17 yrs).

## Methods

This prospective, open, non-controlled, non-interventional, multicenter study was conducted from September 2010 to October 2012 in Germany. Overall 1.482 patients (93 asthmatic children (6.2%), 73 asthmatic adolescents (4.9%)) participated in the study.

The patients rated their symptoms as a combined score of severity (scale: 0 [none] – 3 [severe]) and frequency (scale: 0 [none] – 4 [very often]). In the combined RC score, the severity of rhinitis and conjunctivitis were pooled (scale: 0 [none] – 6 [severe]). In the asthma score, the severity and the frequency were pooled (scale: 0 [none] – 7 [severe]).

## Results

During the season preceding AIT treatment 93/ 92% of children/ adolescents with asthma had used symptomatic medication for RC symptoms. This rate dropped to 64/ 68% (1^st^ season) and to 57/ 41% (2^nd^ season). Likewise the RC score in these patients decreased from 4.06/ 4.13 to 1.86/ 1.82 (1^st^ year) and to 1.33/ 1.59 (2^nd^ year). Also the asthma score was reduced from 3.36/ 3.55 to 1.24/ 1.51 (1^st^ year) to 0.71/ 1.13 (2^nd^ year).

An improvement in health status after two years of treatment was documented by 96/ 96%.

Adverse events occurred in 19.4/ 17.8% over two years of treatment. The incidence of non-fatal serious adverse events was 3.2/ 0.0%.

At the end of the 2^nd^ season, 96/ 96% evaluated the tolerability of the 5-grass pollen tablets as very good or good.

## Conclusion

Based on the study results, AIT with Oralair 5-grass pollen tablets was well tolerated by children and adolescents with asthma in routine medical practice. Symptomatic medication for RC symptoms use was significantly reduced. The asthma- and the RC score were also reduced significantly after one and two years under treatment with Oralair 5-grass pollen tablets compared to the season preceding AIT.

